# UCN2: a new candidate influencing pancreatic β-cell adaptations in pregnancy

**DOI:** 10.1530/JOE-19-0568

**Published:** 2020-02-27

**Authors:** Sian J S Simpson, Lorna I F Smith, Peter M Jones, James E Bowe

**Affiliations:** 1 Department of Diabetes, School of Life Course Sciences, Faculty of Life Science and Medicine, King’s College London, London, UK

**Keywords:** insulin, islet, β-cell adaptation, pregnancy, corticotropin-releasing hormone, urocortin

## Abstract

The corticotropin-releasing hormone (CRH) family of peptides, including urocortin (UCN) 1, 2 and 3, are established hypothalamic neuroendocrine peptides, regulating the physiological and behaviour responses to stress indirectly, via the hypothalamic-pituitary-adrenal (HPA) axis. More recently, these peptides have been implicated in diverse roles in peripheral organs through direct signalling, including in placental and pancreatic islet physiology. CRH has been shown to stimulate insulin release through activation of its cognate receptors, CRH receptor 1 (CRHR1) and 2. However, the physiological significance of this is unknown. We have previously reported that during mouse pregnancy, expression of CRH peptides increase in mouse placenta suggesting that these peptides may play a role in various biological functions associated with pregnancy, particularly the pancreatic islet adaptations that occur in the pregnant state to compensate for the physiological increase in maternal insulin resistance. In the current study, we show that mouse pregnancy is associated with increased circulating levels of UCN2 and that when we pharmacologically block endogenous CRHR signalling in pregnant mice, impairment of glucose tolerance is observed. This effect on glucose tolerance was comparable to that displayed with specific CRHR2 blockade and not with specific CRHR1 blockade. No effects on insulin sensitivity or the proliferative capacity of β-cells were detected. Thus, CRHR2 signalling appears to be involved in β-cell adaptive responses to pregnancy in the mouse, with endogenous placental UCN2 being the likely signal mediating this.

## Introduction

The corticotropin-releasing hormone (CRH) peptide family comprises CRH and the structurally related urocortin peptides (UCN1, UCN2 and UCN3). These neuroendocrine peptides are best known for their involvement in regulating the physiological and behavioural responses to stress, through the cognate G-protein-coupled receptors (GPCRs), CRH receptor 1 (CRHR1) and CRH receptor 2 (CRHR2) (Chen *et al*. 1993, Lovenberg *et al*. 1995, Weninger *et al*. 1999, Bakshi *et al*. 2002), as part of the hypothalamic-pituitary-adrenal (HPA) axis. More recent evidence suggests additional, diverse, extra-hypothalamic roles for these peptides in peripheral organs (Paschos *et al*. 2013, Chatoo *et al*. 2018, Chatzaki *et al*. 2019). Thus, CRH expression has been reported in the adrenal gland and the gastrointestinal tract (Suda *et al*. 1984); UCN1 is expressed in heart, skin and adipose tissue (Kimura *et al*. 2002, Seres *et al*. 2004, Wierzbicka *et al*. 2017); and UCN2 and UCN3 have been detected in peripheral blood cells, skeletal muscle, pancreas and gestational tissues such as foetal membranes and placental villi (Petraglia *et al*. 2010). CRHR1 and CRHR2 are also expressed in a wide range of tissues, including cardiac myocytes, the adrenal gland, adipose tissue, skeletal muscle and skin (Hillhouse & Grammatopoulos 2001), also suggesting physiological roles for the CRH peptide family unrelated to the HPA axis. However, under normal circumstances, levels of the peptides in the peripheral circulation are low (Sasaki *et al*. 1987, Ng *et al*. 2004), suggesting that the peptides may be produced locally to function as autocrine or paracrine agents in tissues where the respective receptors are also expressed (Zouboulis *et al*. 2002, Li *et al*. 2013, van der Meulen *et al*. 2015).

There is increasing evidence that the CRH peptide family may be involved in peripheral metabolic control via direct actions on insulin-secreting β-cells in pancreatic islets of Langerhans (Li *et al*. 2007, Schmid *et al*. 2011). Both CRHR1 and CRHR2 are expressed in rodent (Kanno *et al*. 1999, Schmid *et al*. 2011) and human islets (Amisten *et al*. 2013), whilst *in vitro* administration of exogenous CRH stimulates insulin secretion from mouse and human islets as well as enhancing proliferation in neonatal rat β-cells (Huising *et al*. 2010). Similarly, β-cell-derived UCN3 has been implicated in the local regulation of both insulin and glucagon release (Li *et al*. 2007). Despite the evidence demonstrating direct effects of exogenous CRH on islet function, the physiological relevance of this interaction is unclear, given the islets would not normally be exposed to significant levels of peptides of the CRH family. There is some evidence that placentally derived CRH and urocortins are involved in various biological functions associated with pregnancy (Thomson 2013, You *et al*. 2014). Thus, pregnancy represents one possible physiological state in which the effects of the CRH family on islet function may play a role.

During pregnancy, maternal insulin resistance increases and this is compensated for by increases in β-cell mass and enhanced insulin secretory responses to elevations in plasma glucose (Xue *et al*. 2010, Pasek & Gannon 2013, Baeyens *et al*. 2016). We have recently reported an upregulation of *Crh*, *Ucn2* and *Ucn3* mRNA expression in mouse placenta on gestational day 12 (Drynda *et al*. 2018), which correlates to the initiation of β-cell adaptations in rodent pregnancy (Rieck & Kaestner 2010). Similarly, in human pregnancy, levels of CRH in the peripheral circulation increase as gestation progresses (Campbell *et al*. 1987, Sasaki *et al*. 1987) and CRH immunoreactivity has been reported in human placenta (Grino *et al*. 1987), consistent with a placental source for the circulating CRH. In the current study, we have therefore investigated a potential role for the CRH peptide family in the regulation of glucose homeostasis during pregnancy.

## Materials and methods

### Animals

Female Institute of Cancer Research (ICR) mice (8–12 weeks of age, Envigo, Bicester, UK) were used for *in vivo* studies. This is a commonly used outbred mouse strain with very good reproductive and maternal characteristics. All animals were housed under controlled, pathogen free conditions (12-h light/dark cycle (07:00–19:00 h lights on), temperature 22 ± 2°C) and provided with standard chow diet and water* ad libitum*. For timed pregnancy studies, female mice were mated with male ICR mice and the presence of vaginal plug assessed daily and denoted day 1 of pregnancy if present. Age-matched female mice were used for non-pregnant studies, with procedures carried out at the same time intervals as described for pregnancy studies. All procedures were conducted under approval by King’s College London Animal Welfare and Ethical Review Board and were undertaken in accordance with United Kingdom Home Office Regulations.

### Islet isolation and insulin secretion *in vitro*

For *in vitro* insulin secretion studies, pancreatic islets were isolated from female ICR mice via collagenase digestion of the exocrine pancreas, as described previously (Rackham *et al*. 2016). Isolated islets were subsequently maintained at 37°C in RPMI (Sigma) supplemented with 10% (vol/vol) foetal bovine serum, 2 mmol/L glutamine and 100 U/mL penicillin/0.1 mg/mL streptomycin for 24 h before use. Islets were loaded into a multi-channel, temperature-controlled perifusion system, as described previously (Liu *et al*. 2013), and pre-perifused for 1 h with physiological salt buffer (Bowe *et al*. 2019) containing 2 mmol/L glucose before being exposed to 20 mmol/L glucose in the presence or absence of the CRHR agonists, CRH (50 nmol/L, Sigma), stressin I (100 nmol/L, Tocris) or UCN2 (100 nmol/L, Sigma) at 37°C. Perifusate samples were collected every 2 min and insulin secretion was quantified using an in-house insulin RIA (Jones *et al*. 1988).

### *In vivo* osmotic minipump studies

Osmotic minipumps (ALZET®, Model 1002, Charles River) were implanted subcutaneously into pregnant or non-pregnant mice to chronically administer test agents. Surgical implantation of osmotic minipumps was carried out on day 7 of pregnancy (or equivalent time interval for non-pregnant mice) under isoflurane anaesthesia (Isothesia®, Henry Schein®). Minipumps were loaded with physiological saline, non-specific CRHR antagonist (α-helical CRF_9–41_, 1 mg/ml, Tocris) or receptor-specific CRHR antagonists, antalarmin hydrochloride (1 mg/mL, Tocris) or antisauvagine-30 (3 mg/mL, Tocris) for CRHR1 and R2, respectively. Test agents were delivered at a rate of 0.25 µL/h for a total period of 11 days. Assessment of glucose tolerance and insulin tolerance were conducted on gestational days 16 and 18, respectively.

### Assessment of glucose homeostasis

Intraperitoneal glucose tolerance tests (IPGTT) were conducted on day 16 of gestation. Mice were fasted from 09:00 h for 6 h and then administered with glucose (2 g/kg, Sigma). Blood sampling was performed by small tail prick at time points 0, 15, 30, 60, 90 and 120 min following glucose administration to determine blood glucose levels using an Accu-Chek glucose metre (Roche Diagnostics). Intraperitoneal insulin tolerance tests (IPITT) were conducted on day 18 of gestation. Mice were again fasted from 09:00 h for 6 h prior to metabolic testing and were subsequently administered with insulin (0.75 IU/kg, Sigma). Blood sampling was performed by small tail prick at time points 0, 15, 30, 45 and 60 min following insulin injection to determine blood glucose levels.

### Measurements of circulating CRH-related peptides

On day 18, animals were killed by intraperitoneal injection of terminal anaesthesia (Euthatal®, Merial Animal Health Ltd, Bracknell, UK) and terminal blood samples were collected via cardiac puncture into sterile heparin-coated tubes. Samples were also collected from control pregnant mice on day 16. Samples were centrifuged (1800 ***g***, 20 min, 4°C) and the subsequent plasma was stored at −20°C for later assay of circulating peptide levels using commercially available ELISA kits (CRH: CEA835Mu, Cloud-Clone Corp, Houston, TX, USA; UCN1: CEA231Mu, Cloud-Clone Corp; UCN2: MOFI00425, ELISAGenie, London, UK; UCN3: CED140Mu, Cloud-Clone Corp) following the manufacturers’ instructions.

### Quantification of mRNA expression

Isolated female islets from non-pregnant and pregnant (day 16) mice were immediately snap frozen in liquid nitrogen following purification from the exocrine pancreas for subsequent RNA extraction using RNeasy Mini Kit (Qiagen) and High-Capacity cDNA Reverse Transcription Kit (Applied Biosystems) for cDNA synthesis, as described previously (Drynda *et al*. 2018). Placenta samples were also collected after termination at day 18 of pregnancy and snap frozen. RNA extraction and cDNA conversion were conducted as described earlier. Islet CRH receptor and placental CRH ligand mRNA expression were subsequently quantified by quantitative RT PCR (qRT-PCR) using SYBR Green PCR Kit (QuantiTect, Qiagen) and a LC96 Light Cycler (Roche Diagnostics). QuantiTect primer assays were used for expression analysis of genes of interest using glyceraldehyde 3-phosphate dehydrogenase (*Gapdh*) as the housekeeping gene (Mouse *Crh*-QT01055789, *Ucn1*-QT00326879, *Ucn2*-QT01556534, *Ucn3*-QT00302267, *Crhr1*-QT00106232, *Crhr2*-QT00151543, *Gapdh*-QT01658692, Qiagen).

### Assessment of β-cell mass

For osmotic minipump studies, bromo-deoxy-uridine (BrdU, 1 mg/mL, Sigma) was administered in the drinking water from day 14 to day 18 of pregnancy with fresh BrdU drinking water being replaced every 2 days. After termination at day 18, pancreata were dissected, fixed in 4% paraformaldehyde (Sigma) and embedded in paraffin wax before being cut into 5 µm thick sections using Leica microtome (RM2255). Representative sections (3–4 sections per animal), approximately 150 µm apart, were co-stained with guinea pig anti-insulin antibody (1:200, Dako) to visualise islet β-cells and monoclonal mouse anti-BrdU antibody (1:100, Sigma) to identify proliferating cells as previously described (Bowe *et al*. 2019). Images were taken on Nikon Eclipse TE2000-U fluorescent microscope and quantification of BrdU-positive β-cells and β-cell area was performed using ImageJ 1.49c software.

### Statistical analysis

Statistical analysis was performed using GraphPad Prism 8.0 software. For comparison between two groups, unpaired, two-tailed Students *t*-test was used. For *in vivo* glucose and insulin tolerance tests, two-way repeated-measures ANOVA was used, followed by Tukey’s multiple comparison test to identify the significance between multiple groups.

## Results

### CRH receptor gene expression profile in pregnancy

**Figure 1:**
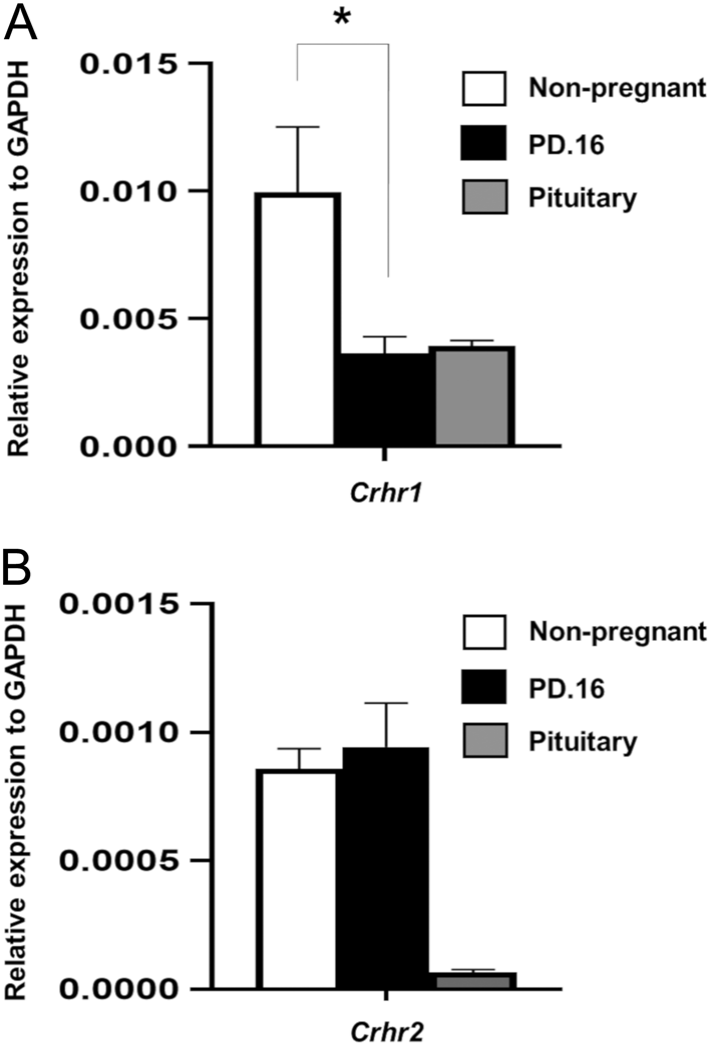
Expression of *Crhr1* (A) and *Crhr2* (B) mRNAs by isolated female islets in non-pregnancy (white bar) and pregnancy day 16 (PD.16; black bar). Anterior pituitary was used as a positive control (grey bar) and mRNA expression levels were quantified to the relative expression of housekeeping gene, *Gapdh*. *Crhr1* mRNA expression levels decreased significantly during pregnancy (~60%), whereas levels of *Crhr2* expression were unchanged. Data are presented as mean + s.e.m., *n* = 5, *P < 0.05; Students *t*-test non-pregnant vs PD.16.

**Figure 2:**
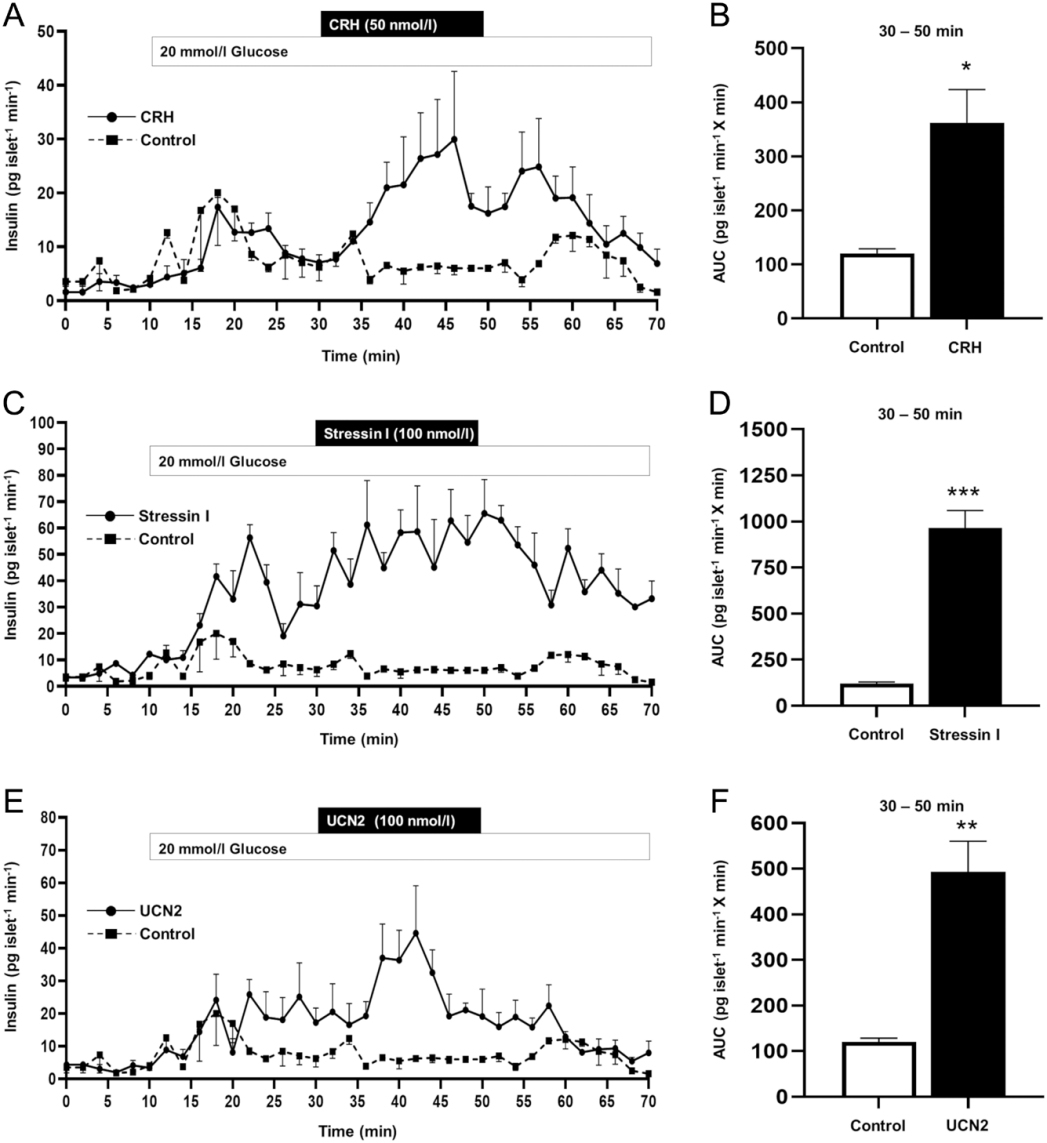
Effect of exogenous CRH (A), CRHR1-specific agonist stressin 1 (C) and CRHR2-specific agonist UCN2 (E) on dynamic insulin secretion from isolated, perifused female mouse islets. Islets were exposed to physiological buffer containing 20 mmol/L glucose only or supplemented with agonists between 30 and 50 min. All CRHR agonists potentiated glucose-stimulated insulin secretion over that seen from control islets, as demonstrated by the rate of insulin secretion (A, C, E) and area under curve data (B, D, F). Data are presented as mean ± s.e.m., *n* = 3–4 per treatment group, AUC 20 mmol/l glucose + agonist, 30–50 min, *P < 0.05, **P < 0.01, ***P < 0.001; Students *t*-test control vs agonist treatment.

Islets isolated from non-pregnant and pregnant (d.16) female mice expressed both *Crhr1* and *Crhr2* mRNAs, as shown in Fig. 1. As expected, *Crhr1* expression in islets was higher than *Crhr2* expression, displaying an analogous expression pattern for the receptors to that in the pituitary, a classical target for CRH. Islet *Crhr1* mRNA expression was significantly reduced during pregnancy compared to non-pregnant levels (Fig. 1A), whereas islet *Crhr2* mRNA levels were unchanged between non-pregnant and pregnant animals (Fig. 2B). Thus, islets express receptors for the entire CRH family of peptides.

### Effects of CRH receptor stimulation on insulin secretion

Activating either CRHR1 or CRHR2 enhanced glucose-induced insulin secretion from isolated mouse islets in a dynamic perifusion system, as shown in Fig. 2. Exposure to 20 mmol/L glucose initiated a rapid increase in insulin secretion, which was further potentiated by the addition of CRH (acting as a non-specific CRHR1 and CRHR2 agonist, Fig. 2A); stressin I (a CRHR1-specific agonist, Fig. 2C); or of UCN2 (a CRHR2 specific agonist, Fig. 2E). Area under the curve quantification of glucose-stimulated insulin secretion (30–50 min) confirms the significant potentiation of insulin secretion in the presence of stimulatory concentrations of glucose, induced by all CRH receptor agonists tested (Fig. 2B, D and F). CRHR agonists had no significant effect on insulin secretion at a sub-stimulatory concentration of glucose (data not shown; 2 mmol/L glucose; control, 0.056 ± 0.010 ng/islet/h vs + 50 nmol/L; CRH, 0.045 ± 0.009 vs + 100 nmol/L; stressin I, 0.034 ± 0.007 vs + 100 nmol/L; Ucn2, 0.053 ± 0.008; mean ± s.e.m., *n* = 9 observations *P* > 0.999). Thus, activation of CRHR1 or CRHR2 potentiates glucose-stimulated insulin secretion from islet β-cells.

### Circulating CRH and urocortin profile during pregnancy

**Figure 3:**
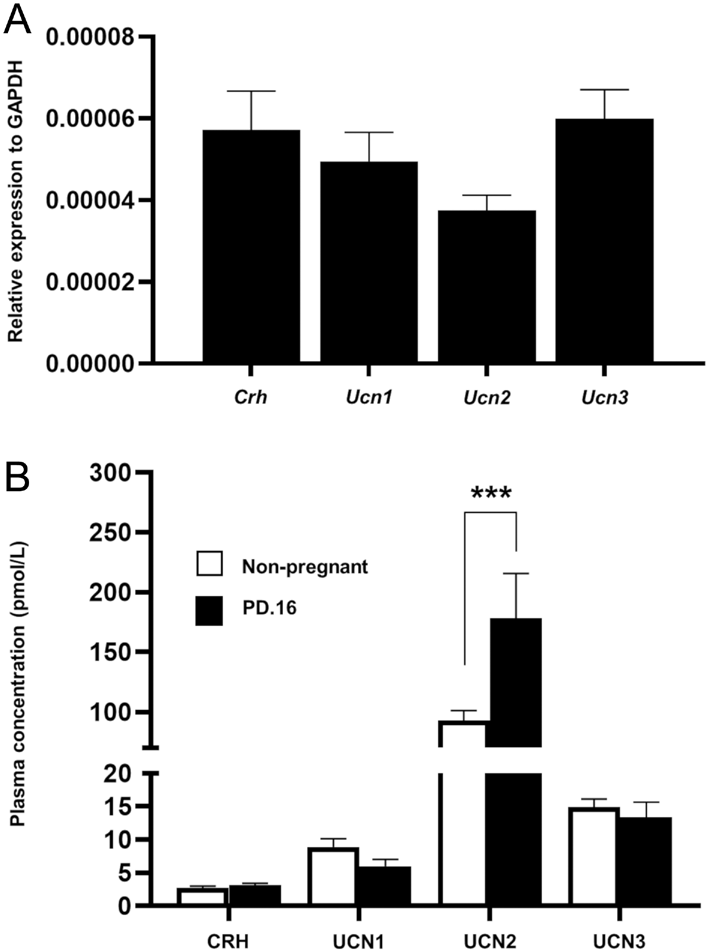
Expression of CRH and urocortins mRNAs in mouse placenta on day 18 of pregnancy (PD.18) (A) and circulating concentrations of CRH peptides during mouse pregnancy (PD.16) (B). Expression levels were quantified to the relative expression of housekeeping gene *Gapdh*. *Crh*, *Ucn1*, *Ucn2* and *Ucn3* mRNAs were all expressed by mouse placenta. Plasma levels of CRH, UCN1 and UCN3 were similar in pregnant and non-pregnant mice. However, plasma UCN2 was significantly elevated during pregnancy. Data presented as mean + s.e.m., *n* = 6, ***P < 0.001; two-way ANOVA followed by Tukey’s multiple comparisons test.

qRT-PCR measurements demonstrated that mRNAs for *Crh*, *Ucn1*, *Ucn2* and *Ucn3* were all expressed by mouse placenta at day 18 at similar levels (Fig. 3A), confirming our previous observations (Drynda *et al*. 2018). Furthermore, all four peptides were detected in the peripheral circulation, with UCN2 being the most abundant circulating CRHR agonist (Fig. 3B). The circulating levels of CRH, UCN1 and UCN3 were unchanged between non-pregnant and pregnant female mice. However, circulating levels of UCN2 were elevated almost two-fold by day 16 of pregnancy when compared to age-matched virgin female controls (Fig. 3B). Thus, the pancreatic islets are likely to be exposed to elevated levels of UCN2 during pregnancy, with the placenta being the most likely source for the increased levels. Therefore, the candidate ligand of the CRH family to play a physiological role in the islet adaptation to pregnancy appears to be UCN2.

### Effect of pharmacologically blocking endogenous CRH receptor signalling during pregnancy

**Figure 4:**
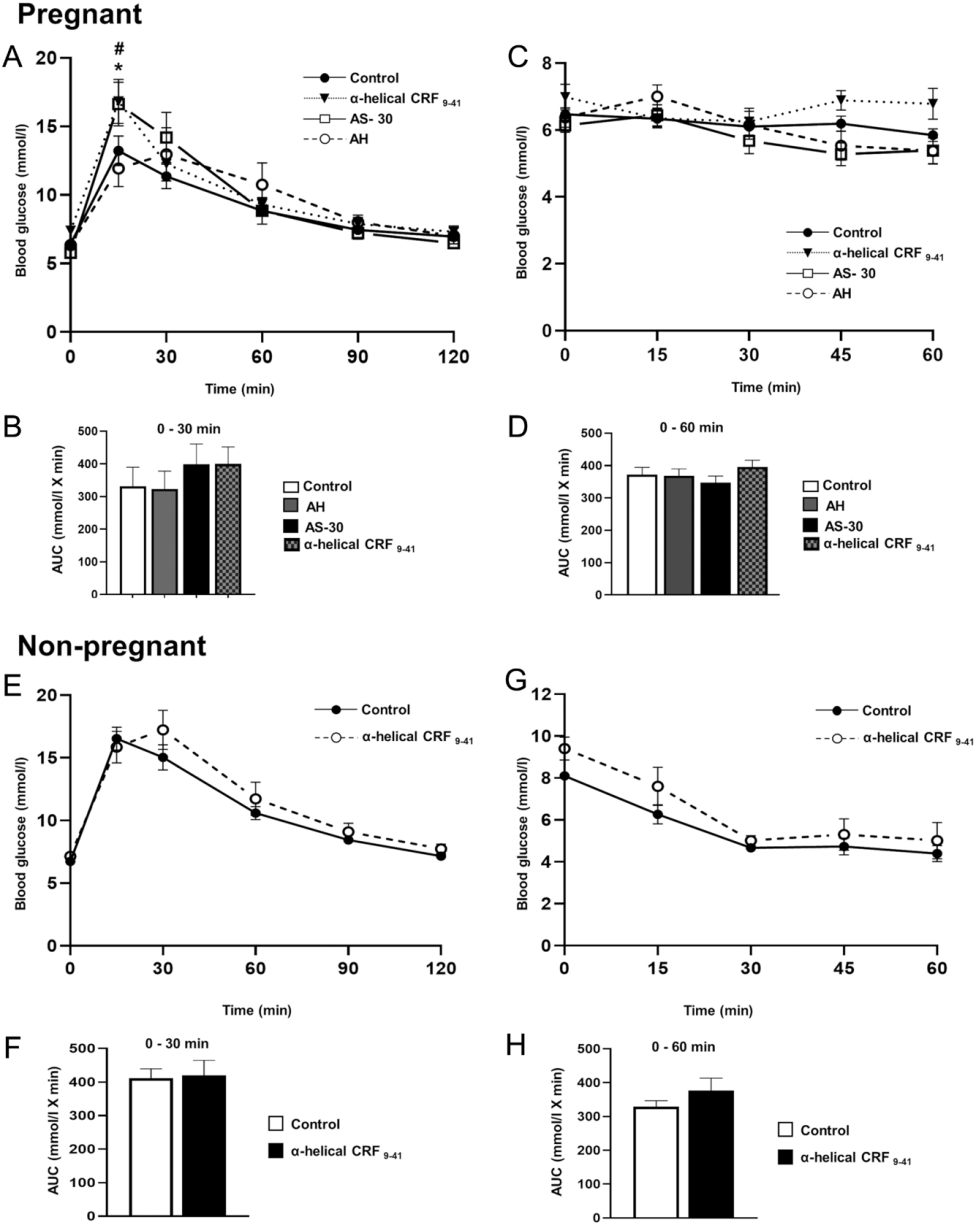
Effects of chronic administration of CRHR antagonists on glucose homeostasis during pregnancy (A, B, C and D) and non-pregnancy (E, F, G and H). Pregnant mice (PD.16) treated with either α-helical CRF_9–41_ or AS-30 (antisauvagine-30) displayed a significant impairment in glucose tolerance 15 min after glucose loading (2 g/kg) when comparison to control mice administered saline (solid black line with solid circles). No difference in glucose tolerance was seen in mice administered AH (antalarmin hydrochloride). AUC from 0 to 30 min for each treatment group is displayed in panel B. No change in overall insulin sensitivity was observed between all treatment groups (C). AUC from 0 to 60 min for each treatment group is displayed in panel D, (*n* = 7–19). In non-pregnant mice chronic administration of α-helical CRF_9–41_ had no significant effects on glucose tolerance (E) or insulin sensitivity (G). AUC for glucose tolerance 0–30 min and insulin sensitivity 0–60 min are displayed in panel F and H respectively, (*n* = 5–6). Data are presented as mean ± s.e.m., # (control vs α-helical CRF_9–41_)/* (control vs AS-30): 15 min *P* < 0.05; two-way repeated measures ANOVA followed by Tukey’s multiple comparisons test.

The consequences of pharmacological blockade of CRH receptor signalling *in vivo* was assessed in both non-pregnant and pregnant mice, revealing a pregnancy- and receptor-specific phenotype, as shown in Fig. 4. As expected, intraperitoneal administration of glucose, elevated blood glucose levels within 15 min in both pregnant and non-pregnant mice (Fig. 4A and E). Chronic pharmacological blockade of total CRHR signalling during pregnancy with a non-selective antagonist, α-helical CRF_9–41_, resulted in a mild impairment to glucose tolerance, with significantly higher blood glucose concentrations at 15 min after glucose administration, compared to saline controls (Fig. 4A). Chronic administration of the CRHR2 antagonist, antisauvagine-30, resulted in a similar impairment to glucose tolerance in pregnant mice, but not in animals treated with the specific CRHR1 antagonist, antalarmin hydrochloride (Fig. 4A and B). These data are consistent with an endogenous ligand, acting via CRHR2, playing a physiological role in maintaining normal glucose tolerance during pregnancy. All pregnant mice were insulin resistant by day 18 of pregnancy as indicated by the failure to respond to exogenous insulin administration and lowering of blood glucose; however, none of the CRHR antagonists had any detectable effects on insulin sensitivity (Fig. 4C and D). Chronic treatment of non-pregnant female mice with α-helical CRF_9–41_ to block total CRHR signalling had no significant effect on glucose tolerance or insulin sensitivity (Fig. 4E, F, G and H). Given the lack of effect of α-helical CRF_9–41_, receptor-specific antagonists were not tested outside of pregnancy. Thus, CRHR2 activation by an endogenous ligand is involved in maintaining glucose homeostasis specifically during pregnancy.

**Figure 5:**
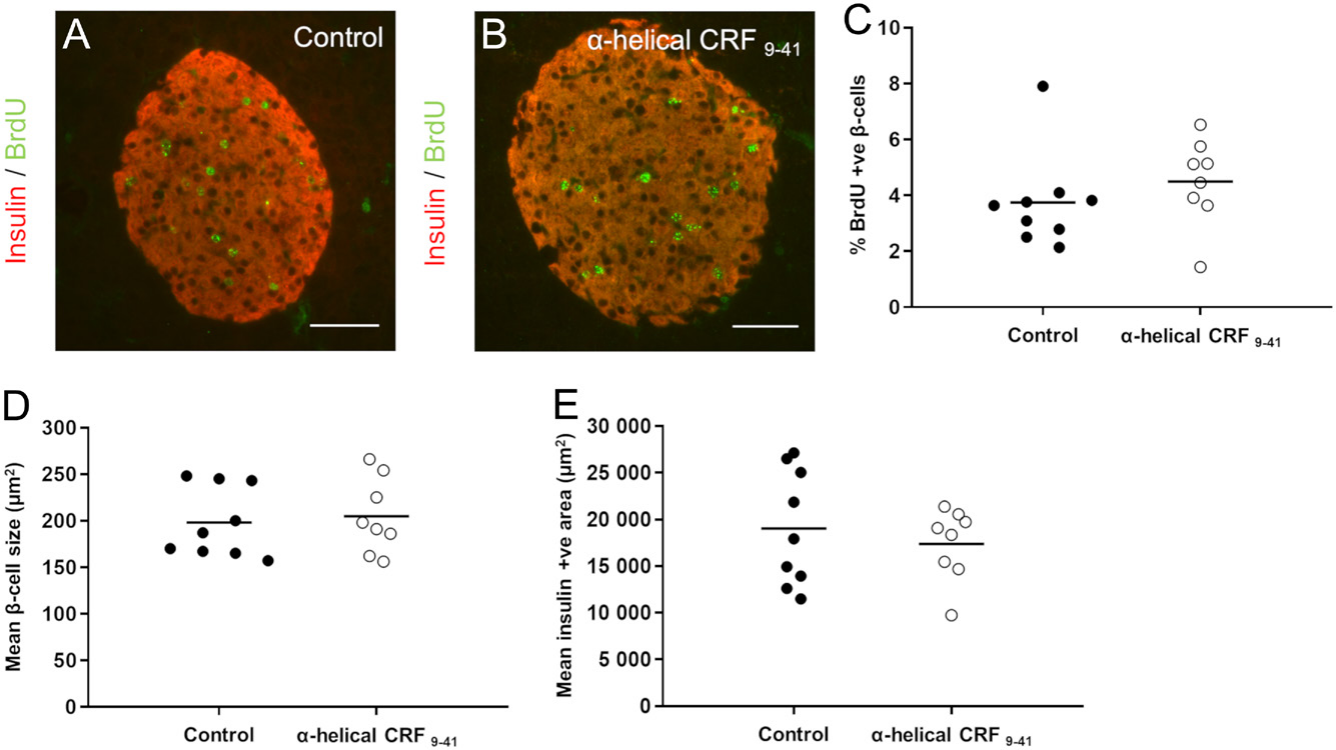
Effect of chronic administration of a non-selective CRHR antagonist (α-helical CRF_9–41_) on β-cell morphology during pregnancy. Representative images of immunostaining for the measurement of β-cell proliferation in control (A) and α-helical CRF_9–41_ (B) islets showing insulin staining (red) and BrdU staining (green). Mice administered BrdU from days 14–18 of pregnancy displayed no significant differences in the percentage of BrdU-labelled β-cells between control and α-helical CRF_9–41_ treated mice (C). Average β-cell size (D) and average β-cell islet area (E) were also unchanged between control and antagonist treatments. Data presented showing quantification (3–4 sections/animal analysed) for individual animals with bar showing mean, *n* = 8–9 animals per treatment group. Scale bar 50 μm.

In addition to effects on whole body glucose homeostasis, pregnancy in mice is also associated with an increased rate of β-cell proliferation to increase the functional β-cell mass (Rieck & Kaestner 2010). This was evaluated by BrdU^+^ β-cell staining (Fig. 5A and B). Chronic blockade of total CRHR signalling during pregnancy using α-helical CRF_9–41_, had no significant effects on β-cell proliferation, β-cell size or the average insulin^+^ β-cell area, as shown in Fig. 5C, D and E. The effects of CRHR activation on glucose homeostasis during pregnancy are therefore most likely direct effects on the β-cell to enhance insulin secretion rather than to increase the β-cell mass.

## Discussion

During pregnancy, the metabolic profile of the mother adapts to ensure a sufficient supply of energy for the developing fetus. A progressive increase in maternal insulin resistance across pregnancy represents a key mechanism for increasing fuel availability to the fetus (Freemark 2006, Newbern & Freemark 2011). This insulin resistance is compensated for by an increase in the maternal functional β-cell mass and enhanced insulin secretory responses (Baeyens *et al*. 2016). Failure of the β-cell to adapt to the maternal metabolic load can lead to maternal glucose intolerance and, eventually, to overt gestational diabetes (Zhang *et al*. 2010, Plows *et al*. 2018). In rodent models, the early β-cell adaptations to pregnancy involve non-placental signals (Drynda *et al*. 2015), but as placentation is established and pregnancy progresses, the placenta becomes an important endocrine organ, secreting numerous hormonal signals, which influence maternal and foetal physiology (Jansson 2016). The lactogenic hormones, prolactin and placental lactogen, are important pregnancy-associated signals, well-established to act via β-cell prolactin receptors to induce β-cell mass expansion and enhance insulin secretion (Brelje *et al*. 1993, Sorenson *et al*. 1993, Vasavada *et al*. 2000, Huang *et al*. 2009). These effects may be mediated, at least in part, by an upregulation of intra-islet serotonin (Kim *et al*. 2010, Ohara-Imaizumi *et al*. 2013). However, the mouse placenta expresses approximately 80 different ligands for which β-cells express the cognate GPCRs (Drynda *et al*. 2018), and it is unlikely that the lactogenic hormones are the only signals involved in regulating islet adaptations. These placental ligands include a number of peptides more usually associated with hypothalamic neuroendocrine functions. We have recently identified kisspeptin as an important placental signal regulating β-cell function during pregnancy (Bowe *et al*. 2019). The current study extends these observations to implicate another classical hypothalamic neuroendocrine system, the CRH peptide family, in placental control of β-cell function.

The expression profile of CRH receptors in mouse islets is consistent with previous reports confirming the expression of both *Crhr1* and *Crhr2* using mouse (Huising *et al*. 2011) or human (Amisten *et al*. 2013) islets. These observations suggest that islet cells have an innate capacity to recognise and respond to circulating CRH and the urocortin peptides. The decreased expression levels of *Crhr1* during pregnancy is also suggestive of a shift in the receptor ratio to potentially direct *Crhr2* signalling under the influence of placental signals. Accordingly, our *in vitro* measurements of insulin secretion from isolated islets, demonstrated that activation of either CRHR1 or CRHR2 significantly potentiates glucose-stimulated insulin secretion (GSIS). Similar to other β-cell GPCRs, activation of CRHR1 and CRHR2 only enhanced insulin secretion in the presence of a stimulatory concentration of glucose, suggesting that the physiological function of receptor activation is to module the extent of the insulin secretory response to elevated glucose concentrations, rather than to initiate secretion. Our dynamic measurements of insulin secretion from isolated islets correspond with studies using mouse or human islets in static incubations (O’Carroll *et al*. 2008, Huising *et al*. 2010) and imply that increased levels of CRHR agonists will result in an enhanced glucose-induced insulin secretory response. However, whilst previous studies have suggested a role for the CRH family in regulating islet function, the physiological purpose of this effect was unclear.

Placental expression and secretion of CRHR agonists is contentious. Earlier studies detected CRH mRNA and immunoreactivity in placentae from humans and non-human primates (Sasaki *et al*. 1987, Frim *et al*. 1988, Robinson *et al*. 1989), but failed to detect it in non-primate species including lemur, guinea pig and rat (Robinson *et al*. 1989). In human pregnancy, levels of CRH in the peripheral circulation increase as gestation progresses (Campbell *et al*. 1987, Sasaki *et al*. 1987). It has thus been suggested that the physiological purpose of this increase is in regulating parturition through modulation of signals controlling myometrium contractility and inflammation (McLean *et al*. 1995, Thomson 2013, You *et al*. 2014). Contrary to human pregnancy, placental CRH in rodents is not thought to have a significant role in initiating parturition, with evidence of a more influential role in facilitating implantation particularly during murine pregnancy (Athanassakis *et al*. 1999). Increased expression of UCN2 mRNA and protein has been reported in both human and mouse gestational tissues (including foetal membranes, myometrium and placenta) (Voltolini *et al*. 2015), although conflicting reports suggest no significant change in circulating levels of UCN1, UCN2 or UCN3 during human pregnancy (Pepels *et al*. 2010). In the current study we detected the expression of mRNAs for all members of the CRH family in mouse placenta. Circulating levels of CRH, UCN1 and UCN3 were unchanged in pregnant and non-pregnant mice, suggesting that these ligands are not released by the mouse placenta at significant levels, however circulating levels of UCN2 were significantly increased during gestation. The circulating concentrations of UCN2 which we detected during pregnancy are close to the reported EC50 values for CRHR2 (Hauger *et al*. 2003, Dautzenberg *et al*. 2004, Patel *et al*. 2012) and are consistent with β-cell CRHR2 activation in response to pregnancy signals. These observations are also consistent with the placenta being the source of the increased circulating UCN2 during mouse pregnancy, analogous to the increases in placentally derived kisspeptin in the circulation during mouse and human pregnancy (Dhillo *et al*. 2006, Mark *et al*. 2013, Bowe *et al*. 2019) and suggest that it may potentially play a physiological role during pregnancy. However, it cannot be ruled out that the pregnancy-associated UCN2 derives from an alternative peripheral source, such as skin or skeletal muscle where it is also highly expressed (Chen *et al*. 2004).

Irrespective of its source, our *in vivo* studies suggest a role for circulating UCN2 in the regulation of β-cell insulin secretory responses during mouse pregnancy. Thus, pharmacological blockade of CRHR2 impaired glucose tolerance in pregnant mice, but a similar impairment was not observed with CRHR1 blockade, nor in non-pregnant females. The lack of effect of *in vivo* CRHR blockade on insulin resistance during pregnancy suggests that the impaired glucose tolerance reflects a β-cell targeted effect, consistent with our *in vitro* observations of enhanced insulin secretion in response to CRHR2 activation. Most placental hormones involved in β-cell adaptations to pregnancy exert dual effects to acutely increase the rate of insulin secretion from individual β-cells, and chronically to induce expansion of the functional β-cell pool. These compensatory mechanisms ensure that the mother can sustain a robust insulin secretory response to elevated plasma glucose, especially in the prevailing insulin resistant environment. Under normal circumstances the rate of β-cell proliferation is very low, but chronic exposure to lactogenic hormones (Brelje *et al*. 1993, Huang *et al*. 2009, Baeyens *et al*. 2016) or to kisspeptin (Bowe *et al*. 2019) during gestation increases the rate of β-cell proliferation, and so increases the functional β-cell mass both *in vitro* and *in vivo*. In the current study, chronic blockade of total CRH receptors during pregnancy had no significant effects on β-cell size or proliferation, or on the overall β-cell mass. This provides further evidence that the impairment to glucose tolerance *in vivo* during pregnancy is due to an endogenous ligand, specifically targeting CRHR2, enhancing β-cell insulin secretion. The physiological significance of these differences in modes of action of placental factors is uncertain, but there may be therapeutic advantages in the ability of UCN2 to enhance glucose-induced insulin secretion without targeting the clinical challenges of manipulating β-cell proliferation.

The variability of maternal glycaemia throughout pregnancy can range from normal/mild glucose intolerance, to severe in the case of gestational diabetes. The pharmacological blockade of CRHR2 signalling during pregnancy appears to reveal a transient and mild glucose intolerance in comparison to the more profound defect in glucose tolerance displayed by mutant PRLR mice (Huang *et al*. 2009). Given the importance of maintaining appropriate maternal glycaemic control during pregnancy, it is perhaps not surprising that there are multiple control mechanisms that ensure an integrated β-cell insulin secretory response. Therefore, the mild phenotype displayed may have been compensated by complementary signals to prevent major disruptions to glucose homeostasis.

In summary, we have demonstrated that CRHR2 signalling is involved in β-cell adaptive responses to pregnancy in the mouse, with endogenous placental UCN2 being the likely signal mediating this adaptation. Unlike other identified placental signals, the effects of UCN2 appear to be confined to amplifying glucose-induced insulin secretion without concomitant alterations in the β-cell mass. Blocking the endogenous CRHR2 agonist during gestation induces a mild glucose intolerance rather than overt gestational diabetes suggesting that UCN2 may act in concert with other placental signals to fine-tune the compensatory β-cell adaptations to maternal insulin resistance during pregnancy. Deciphering the interplay between these different signals will lead to a more comprehensive understanding of the pathophysiology of gestational diabetes and may offer novel diagnostic or therapeutic strategies.

## Declaration of interest

The authors declare that there is no conflict of interest that could be perceived as prejudicing the impartiality of the research reported.

## Funding

This work was supported by grant funding from both the Diabetes Research and Wellness Foundation (DRWF) (SCA/OF/12/18) and the Medical Research Council (MRC) Doctoral Training Program studentship (Sian J S Simpson).
